# Basal Cell Carcinoma of Hand Presenting With Severe Sepsis

**DOI:** 10.7759/cureus.42072

**Published:** 2023-07-18

**Authors:** Pallavi R Ganesan, Paul J Hannon

**Affiliations:** 1 Department of Research, Alabama College of Osteopathic Medicine, Dothan, USA; 2 Department of Radiology, Mobile Infirmary Medical Center, Mobile, USA

**Keywords:** anemia, cutaneous oncology, hand cancer, sepsis, basal cell carcinoma in situ

## Abstract

Basal cell carcinoma (BCC) is one of the most common skin malignancies in the United States. It commonly presents in the neck and head as a papulo-nodular lesion that is slow-growing with rare metastasis. BCC has a high curative rate often with Mohs surgery or excision of the lesion.

In this case, we present an 80-year-old male who presented to the emergency department with a necrotizing wound on his left hand and met sepsis criteria. His necrotizing wound was identified as severe BCC of his hands which he has a 10-year history diagnosis of. He received appropriate sepsis measures with fluids, antibiotics, and blood transfusions but denied any further care for his BCC.

Our case is unique because hands are an extremely uncommon presentation location of BCC. It is also unique because he presented as an extremely aggressive case while most cases of BCC are relatively innocuous. Early management of BCC is important, especially with a multidisciplinary approach as it can significantly reduce mortality risk.

## Introduction

Basal cell carcinoma (BCC) is one of the most common malignancies in the United States with an increase in annual incidence by 4%-8%. The malignancy is commonly caused by cumulative sun exposure, especially in the aging population. [1] The development of BCC depends on a variety of factors including previous environmental exposures, genetic predisposition, and phenotypical differences. But the most commonly accepted risk factor is exposure and susceptibility to ultraviolet radiation (UVR).

BCC presents clinically as a papulo-nodular lesion with classical rolled borders and telangiectasias with or without ulceration [2]. It commonly presents on the head or neck skin 70% of the time. Histologically, it is characterized as proliferating keratinocytes from basal cells of the epidermis. BCC is well known for its relatively innocuous course with slow growth and mainly local extension with a low likelihood of metastasis [2]. It is usually diagnosed based on clinical presentation and biopsy. Treatment is usually through curative excision or Mohs surgery.

Here, we present a case of a male presenting with an aggressive necrotic BCC of the hand which met sepsis criteria in the emergency department. The intent in presenting this case is to emphasize the destructive nature of BCC when untreated as well as highlighting a very rare anatomical presentation.

## Case presentation

The patient is an 80-year-old Caucasian male who presented to the emergency department with a complaint of left-arm pain and weakness. The patient has had multiple admissions at different hospitals over the past three months for severe anemia due to blood loss from the left arm. The patient was diagnosed with BCC on his left hand and wrist 10 years ago. At that time, he denied the Mohs surgery or any further interventions.

The patient states that there has been increased bleeding from his wound which required multiple blood transfusions for anemia within the past few months. Most recently, he reports that he received a transfusion a few days ago for a hemoglobin level of 6.7 g/dL. However, he continued to bleed at home with increasing hand weakness. He described the pain in his left hand as uncontrolled and at a pain level greater than 10/10. The pain did not improve on multiple painkillers and only responded to Fentanyl.

The patient has no other medical history besides the BCC of his left hand for the past 10 years. His surgical history included past left wrist skin cancer excision. His current medications include Acetaminophen 650 mg QD, ascorbic acid 250 mg QD, ferrous sulfate 65 mg QD, oral probiotic complex QD, and Morphine sulfate 7.5 mg PRN. He denies any alcohol or tobacco use. His family, social and drug history is unremarkable.

Notable physical examination findings include a pale-appearing, cachectic male with an edematous left hand and digits. Heart rate and rhythm were regular with no discernable murmurs, gallops, or rubs. The patient refused to remove any dressing on his left hand for a complete evaluation of the wound. The emergency department workup consisted of the following: complete blood count (CBC), complete metabolic panel (CMP), x-ray of extremities, and EKG. The pertinent lab values are in Table 1.

**Table 1:** Pertinent lab findings BUN = blood urea nitrogen, WBC = white blood cell count

Lab Results	Value	Ranges
Calcium	8.4 mg/dL	8.5-10.5 mg/dL
Anion Gap	16 mmol/L	5 -15 mmol/L
BUN	29 mg/dL	5-25 mg/dL
Creatinine	1.20 mg/dL	0.67-1.17 mg/dL
Hemoglobin	6.5 g/dL	12.5 -17.5 g/dL
Hematocrit	21%	39%-53%
WBC	24.7 mm^3^	5-11.5 mm^3^

**Figure 1:**
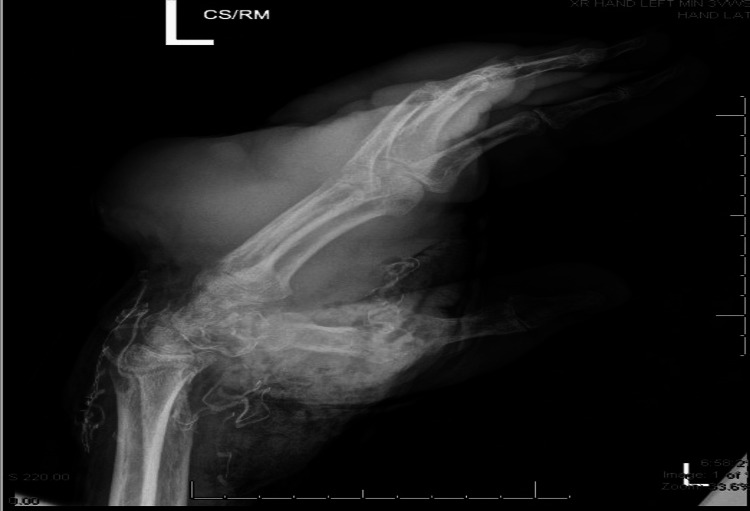
X-ray of right wrist

On CMP, patient had elevated anion gap and decreased calcium. CBC showed low Hg, low RBC count, elevated WBC count and low hematocrit. BMP showed elevated glucose, elevated BUN and creatinine. EKG showed sinus tachycardia of 142 beats per minute. A left-hand x-ray was done which showed an extensive mass throughout entirety of hand. There is high density of serpiginous material throughout region of wrist, likely dressing. There is extensive gas throughout the base of the hand and wrist that has progressed from last known imaging. No fracture or malalignment can be seen in Figure 1.

On admission, the patient met severe sepsis criteria with a BP of 73/29, HR of 124, WBC of 24.6 K/µL with an infectious source likely from the necrotic wound infection of his left hand. The patient received 1 liter bolus 0.9% NaCl fluid as well as 1 g of IV Vancomycin and 2 g IV Ceftazidime (Fortaz). Blood cultures and wound cultures were drawn. There was no growth on blood culture. Additionally, the patient was anemic with his hemoglobin/hematocrit being 6.5/21, respectively, thus he received three units of packed red blood cells (PRBCs).

The patient became relatively stable after the interventions. After the patient was no longer septic, a discussion with the patient and family occurred about further management of the necrotic BCC on his left hand due to concern of increased risk of recurrent sepsis episodes. Multiple physicians overseeing the care recommended amputation of the wrist to prevent further damage along with adjuvant chemotherapy.

However, the patient and wife refused any orthopedic or surgical intervention including amputation of the hand. The patient wanted to only continue treatment for infection with oral antibiotics and a blood infusion for his fatigue. A decision to have a palliative care consult was made for pain control as well as a home health/hospice consideration.

## Discussion

BCC has a 20% lifetime risk of development and is known to be slow growing [1]. There is a generally favorable prognosis as complete surgical excision is almost always curative [2]. The most common risk factor is exposure and susceptibility to UVR [2]. There are multiple studies that have shown that patients with Fitzpatrick type I or type II skin types have a risk two to four times of developing BCC [3].

When assessing an individual’s risk of BCC, the physician should be assessing the number of sunburns and levels of cumulative UVR especially if it occurs in childhood or adolescence. The use of indoor tanning bed use is another dose-related risk factor for earlier-onset BCC [2]. Acute intermittent exposure to UVR, especially during childhood or teenage, is associated with a higher BCC risk during a lifetime. Ionizing radiation can also lead to a higher risk of BCC, mainly at the site of exposure [4]. Other environmental risk factors include repeat micro-injury, scars, chronic ulcers of lower limbs, and prolonged exposure to chemical agents [4].

Primary tumors are approximately less than 2 cm on average. The most common sites include the head and neck in approximately 64% of the population and the trunk in 21% [2]. In a trial [5], they identified that the lowest relative tumor densities involve the foot, thigh, and hand. The hand, which is of interest in this case, is not a common site of BCC even though these areas experience chronic/intermittent exposure to sunlight, chronic trauma, and sunburns. It is suspected that it might be because the palmar hand lacks pilosebaceous glands while the dorsum of the hand has fewer pilosebaceous glands compared to the rest of the body [6]. Another hypothesis is that the hand has a greater thickness of the epidermis and since BCC originates from basal cells in deep layers of the epidermis, there is a decreased occurrence [5]. There is still a lack of clear understanding of why this happens.

When suspecting an aggressive BCC phenotype, there are several factors that are associated with poor outcomes according to a review by Walling [2]. The risk factors include large size, neglect, or longstanding duration (greater than five to 10 years to treatment), deep invasion, and inadequate treatment [2]. In this case, the patient met all the risk factors for the classification of aggressive BCC.

Metastatic BCC is extremely rare with an estimated incidence of 0.00028%-0.55%. The most common sites of metastasis in descending order are lymph nodes (53%), lungs (33%), and bones (20%) [7].

The presumptive diagnosis of BCC is based on the physician’s interpretation of clinical presentation such as clinical appearance, anatomic location, and patient-reported history. The diagnosis of BCC is confirmed through biopsy. The recommended techniques to be used are punch biopsy, shave biopsy or excisional biopsy [1] There is a varying range of therapies for the treatment of BCC. However, the current cornerstone of BCC treatment is standard excision, Mohs micrographic surgery (MMS) or Curettage and electrodessication (C&E). It is to the discretion of clinician on technique used as there are many factors involved in decision-making [1]. Other non-surgical treatments included cryosurgery or photodynamic therapy. It is very important when assessing the efficacy of treatment to have an adequate length of follow-up. It is recommended that once BCC has been diagnosed, a follow-up in-office screening should be done yearly [1].

Our patient met the multiple criteria of aggressive BCC with an enlarged size, long-standing neglect, suspected deep invasion and inadequate treatment [2]. The patient was advised throughout multiple areas of care that lack of treatment can lead to recurrent presentations to the ED. However, he was lost to follow-up from initial diagnosis for approximately 10 years for treatment. Due to the patient's advanced age and aggressive nature at this presentation, the best recommendation was palliative care and home health consultations. Although we were not able to provide curative treatment for this patient, we hope this case serves as a reminder to clinicians that even the most innoculous condition can cause severe presentations if not properly managed. 

## Conclusions

In this case report, we discuss a patient with a long-standing BCC diagnosis of his hands. The case remains unique because the patient presented with an aggressive phenotype of BCC in a very rare location. There was symptomatic care provided to the patient however, the patient ultimately decided on no further treatment at this time. We hope that this case can be added to the literature about BCC presentations in the hand as well as serve to remind patients and physicians that a curative condition can still lead to severe presentations.
